# Gleanings from Our Exchanges

**Published:** 1873-09-15

**Authors:** 


Slowing of the Pulse after Labor.—The retarded pulse which follows parturition has been remarked by McClintock, Blot and Marey, and more recently by Prof. Emilio Falaschi, of Sienna, who contributes to *Lo Sperimentale*, of March, the results of one hundred and eight observations in the hospital of that city, in which he has paid special attention to the age of the patient, her state—whether primiparous or multiparous, the presentation of the fœtus, duration of labor, etc., etc.

He determines that retardation of the pulse is met with in a sensible and persistent degree in only about the fourth of those recently delivered. The number of pulsations varies in each case from 44 to 52; in one case becoming as low as 38.

This slowing, with certain exceptions, shows itself nearly always within eighteen to thirty hours after accouchement, and ceases ordinarily at the fifth or sixth day from the same time. The age of the woman seems to exert a certain influence, the pulse remaining quicker in the young than in those who have passed thirty-five years. Primipara manifest this symptom more frequently than multipara. Neither the season, hour of the day or night, duration of labor, nor presentation and position of the fœtus have any influence in modifying it.

Slowing of the pulse takes place not only after simple labor, but also after the birth of twins; not only after labor at term, but also—according to a few observations—after premature labor at the fourth and sixth months, and abortion before the fourth month. The phenomenon followed, as well, expulsion of fœtuses which had died four or five days previous to birth; but in these cases Sig. Falaschi has never observed it to occur previous to the birth. Whenever accouchement is complicated with considerable hæmorrhage the retardation of the pulse fails to take place, or lasts for so short a period as not to deserve mention.

The labor pains have no influence upon the number of pulsations, at least not when they are not very intense; and the lacteal secretion is also without effect if the slowing of the beats has preceded its establishment. If, however, it is devoloped before this phenomenon occurs, the latter is prevented.

Whenever it is well marked, and when it persists for several hours, it is to be taken as a very favorable sign.—*Medical Record.*

Chronic Tetanus Treated by Excision of Cicatrix.—Mr. Annandale gives a short account of the following case: A boy, aged sixteen wounded his foot. On the ninth day afterwards trismus came on; and he came into hospital on the sixteenth day after the wound, on the seventh of the trismus. There was no laryngeal symptom nor opisthotonos. The patient swallowed well; his bowels were freely opened by croton oil, and for three days he was kept under the influence of small doses of morphia. For two days more he had ninety grains of chloral daily. On the twelfth day of the tetanus, slight contraction of the muscles of the limb began. On the next day, the cicatrix and a piece of nerve which appeared congested were cut out, and all medical treatment stopped. There was no improvement for two days; but on the third day, or sixteenth of the disease, he began to improve, and went out soon, quite well.—*Brit. Med. Jour.*, July 26, '73.

Belladonna in Intestinal Invagination and Hernia.—M. Gallicie (*La France Medicale*), in a paper on belladonna, says it is the special medicament for intestinal invagination, strangulated or not, as also for strangulated hernia. It acts on both the spasmodic and inflammatory elements. In both cases, however applied, its first effect is to alleviate the intensity of pain, and to diminish and arrest the vomiting.

One profession (says *Punch*) is safe from the invasion of woman. She may enter the army, but it is impossible that she can man the navy.

### Gleanings from Our Exchanges.

THE RESUSCITATION OF NEW-BORN CHILDREN.—In *The Clinic* of July 26, '73, is a case republished from the *Medical Press and Circular*, reported by Dr. John J. Marshall, where this gentleman succeeded in resuscitating an apparently lifeless new-born child, after plying artificial respiration, chiefly by the *mouth-to-mouth* method, for a period a little less than four hours.

The case is interesting and instructive, and calls up some cases reported by myself in a paper entitled the "Mouth to Mouth *vs.* the Marshall Hall Method in Asphyxia-Neonatorum," published in the *Lancet and Observer*, No. 1, 1860. The cases were adduced in attestation of the importance of perseverance in artificial respiration, and of the superior efficacy of the mouth-to-mouth method in cases where children born apparently lifeless may yet be susceptible of resuscitation.

In three of my cases the infants were to all appearance dead; but careful examination revealed the continuance of feeble cardiac contractions. In one case it is mentioned "after persevering for several minutes there was a feeble respiratory gasp; then, after a considerable interval, another gasp. These respiratory acts continued to be repeated at long intervals, artificial respiration in the meantime being kept up, and the circulation thereby maintained. An hour passed before the child could maintain its respiration unassisted."

In another case, "the heart was still pulsating, though so feebly as to be just perceptible, and so slowly that the lengthened interval excited my apprehension that each beat would be the last. With the loss of as little time as possible I commenced artificial respiration (by the usual method) the effect of which was soon manifested on the circulation; the action of the heart was increased until it came up to the standard of a healthy breathing infant; the surface changed from pallor and lividity to the healthy complexion. But it seemed that vitality was to be maintained only so long as I persevered in inflating the lungs, for on suspending the operation there was no spontaneous respiratory effort, and the circulation became speedily feeble. However, after keeping up the process at least half an hour I was greeted with a respiratory gasp. There was a recurrence of gasps, at first at long intervals, and afterwards more frequently, until respiration was established."

In a third case, "*at least an hour elapsed before the child gave a gasp*, and two hours before it could be left to do its own breathing. My dependence was upon the *mouth-to-mouth* process. By it I found no difficulty in controlling the circulation. It seemed as though the heart's action might have thus been maintained indefinitely. The 'Marshall Hall method' was tried, but the results were negative; under it pallor and lividity would return to the surface, and the circulation grew gradually more and more feeble, until the heart's action would plainly have soon ceased had it not been aroused by a more ready and efficient method. Several times did I alternate the new method with the old, and just as often did I witness the same striking contrast of phenomena."

Two more cases are instanced in which the asphyxia was profound, showing failure of the Marshall Hall and success of the mouth-to-mouth procedure.

These are types of cases of which I have since seen many. Nor can I better express my confidence in the efficacy and superiority of artificial respiration by the mouth-to-mouth method in asphyxia of new-born children than by quoting from the paper referred to: "I believe it is adequate to resuscitate wherever resuscitation is possible. Give me a beating heart, however feeble and slow its action, and I expect to resuscitate the child; but if motion there has absolutely ceased, I am impressed that the best exertion in the use of any means would be in vain.—A. T. Keyt, M.D., in *The Clinic* for Aug. 23d.

Specialties.—Dr. Robert Barnes says, "I have recently been honored by a visit from a lady of typical modern intelligence, who consulted me about a fibroid tumor of the uterus; and lest I should stray beyond my business, she was careful to tell me that Dr. Brown-Sequard had charge of her nervous system; that Dr. Williams attended to her lungs; that her abdominal organs were intrusted to Sir William Gull; that Mr. Spencer Wells looked after her rectum; and that Dr. Walshe had her heart. If some adventurous doctor should determine to start a new specialty, and open an institution for the treatment of diseases of the umbilicus—the only region which, as my colleague, Mr. Simon, says is unappropriated—I think I can promise him more than one patient."—*London Lancet.*

---

State of Illinois Institution for the Education of Feeble Minded Children.—*Superintendent's Office, Jacksonville, Ill.*—The next term will commence on Wednesday, Sept. 17, 1873. Pupils from Illinois receive board and tuition free. Pupils are expected to be brought to the Institution promptly on time, but not before that time. We have no accommodations for friends of pupils at the Institution, but there are good hotels in town. Street railroad runs from the Junction Depot to the Public Square, and from the Public Square to the Institution.

C. T. WILBUR, M.D., *Supt.*

